# Serum amyloid A binds to fibrin(ogen), promoting fibrin amyloid formation

**DOI:** 10.1038/s41598-019-39056-x

**Published:** 2019-02-28

**Authors:** Martin J. Page, Greig J. A. Thomson, J. Massimo Nunes, Anna-Mart Engelbrecht, Theo A Nell, Willem J. S. de Villiers, Maria C. de Beer, Lize Engelbrecht, Douglas B. Kell, Etheresia Pretorius

**Affiliations:** 1 Department of Physiological Sciences, Stellenbosch University, Stellenbosch Private Bag X1 Matieland, 7602 Stellenbosch, South Africa; 2 Department of Internal Medicine, Stellenbosch University, Stellenbosch, South Africa; 3 Department of Physiology, Saha Cardiovascular Research Center and Barnstable Brown Diabetes Center, University of Kentucky, Lexington, KY USA; 4 Fluorescence Microscopy Unit, Central Analytical Facilities, Stellenbosch University, Stellenbosch, South Africa; 5 School of Chemistry, The University of Manchester, 131 Princess St, MANCHESTER M1 7DN, Lancs, UK; 6 The Manchester Institute of Biotechnology, The University of Manchester, 131 Princess St, MANCHESTER M1 7DN, Lancs, UK; 7 Present Address: Department of Biochemistry, Institute of integrative Biology, Biosciences Building., University of Liverpool, Crown St, Liverpool, L69 7ZB UK

## Abstract

Complex associations exist between inflammation and thrombosis, with the inflammatory state tending to promote coagulation. Fibrinogen, an acute phase protein, has been shown to interact with the amyloidogenic ß-amyloid protein of Alzheimer’s disease. However, little is known about the association between fibrinogen and serum amyloid A (SAA), a highly fibrillogenic protein that is one of the most dramatically changing acute phase reactants in the circulation. To study the role of SAA in coagulation and thrombosis, *in vitro* experiments were performed where purified human SAA, in concentrations resembling a modest acute phase response, was added to platelet-poor plasma (PPP) and whole blood (WB), as well as purified and fluorescently labelled fibrinogen. Results from thromboelastography (TEG) suggest that SAA causes atypical coagulation with a fibrin(ogen)-mediated increase in coagulation, but a decreased platelet/fibrin(ogen) interaction. In WB scanning electron microscopy analysis, SAA mediated red blood cell (RBC) agglutination, platelet activation and clumping, but not platelet spreading. Following clot formation in PPP, the presence of SAA increased amyloid formation of fibrin(ogen) as determined both with auto-fluorescence and with fluorogenic amyloid markers, under confocal microcopy. SAA also binds to fibrinogen, as determined with a fluorescent-labelled SAA antibody and correlative light electron microscopy (CLEM). The data presented here indicate that SAA can affect coagulation by inducing amyloid formation in fibrin(ogen), as well as by propelling platelets to a more prothrombotic state. The discovery of these multiple and complex effects of SAA on coagulation invite further mechanistic analyses.

## Introduction

Serum amyloid A (SAA) refers to a highly conserved family of apoproteins that are synthesised predominantly by the liver^1^ and are transported in the circulation, mainly associated with high-density lipoprotein (HDL)^2^. Their description nearly 40 years ago was the result of analyses of amyloid A (AA) fibrils that allowed for the identification of the precursor SAA apolipoprotein^3^. During inflammatory processes, cytokines induce hepatic SAA synthesis. The secreted SAA associates with circulating HDL and the plasma concentration can increase 1000-fold or more, to levels exceeding 1 mg.mL^−1^ ^4^. SAA profoundly alters HDL composition and structure, with implications for the dynamics of the lipid and apolipoprotein components that constitute the HDL particle^5^. SAA, either produced locally (e.g. in the gut epithelium or by resident macrophages) or transported to sites of inflammation, also forms part of the innate immune system where it activates the inflammasome cascade, leading to immune activation and immunomodulation^6^. It is this proinflammatory function of SAA that could explain the strong relationship between SAA levels and future cardiovascular events^7,8^. Indeed, a question is whether increased levels of circulating SAA promote a prothrombotic state in conditions such as acute coronary syndromes^9^.

Recently we reported that during inflammation, likely due to the presence of highly substoichiometric amounts of lipopolysacharide (LPS) and the broadly equivalent lipoteichoic acids (LTA)^10^, plasma fibrinogen molecules become amyloidogenic, and are associated with an enhanced prothrombotic state^9–11^. The amyloidogenic potential of fibrinogen became apparent with the description that rare sequence variants of fibrinogen in the A alpha-chain (AFib) can deposit as amyloid fibrils, resulting in predominantly renal amyloidosis^12^. Amyloidogenesis is the result of misfolding of precursor proteins with uncoiling of alpha helices and increases in β-sheet structure^13,14^. These misfolded protein structures likely lead to functional effects including a tendency to promote thrombosis^9^.

Fibrinogen and SAA are both acute phase proteins^15^. SAA is also a highly fibrillogenic molecule^16^ and chronically elevated levels may cause reactive systemic amyloidosis (AA type). High plasma concentrations of SAA can result in aggregation as amyloid in β-sheet fibrillar deposits^17^. It is possible that both SAA and fibrinogen could co-deposit in such fibrils.

Although it is well-known that SAA is an excellent biomarker for inflammation, little is known about its potential to induce amyloid changes in fibrin(ogen), which could ultimately promote hypercoagulation and abnormal clotting. Platelets, erythrocytes (RBCs) and circulating plasma molecules all interact and play a fundamental role in normal haemostasis and blood clotting, and in the presence of inflammation can undergo inflammatory changes themselves^18–21^. Since fibrinogen can also interact with other amyloidogenic proteins such as Alzheimer’s disease peptide beta-amyloid^22–24^, the aim of this paper was therefore to examine the amyloidogenic propensity of free SAA when interacting with fibrin(ogen) in the blood of healthy individuals, and in a purified fibrinogen model. Further to this, SAA has also been shown to bind to platelets^25^ and impact platelet activation^26^. We also explored the effect of SAA on human fibrin clot properties, as well as platelet function and morphology.

## Materials and Methods

### Blood samples

Healthy female donors (N = 21), aged 29 to 59, were recruited for this *ex vivo* study (see Table 1 for demographic data). Exclusion criteria were known acute and chronic inflammatory conditions, smoking, and contraceptive or hormone replacement treatment. To prevent gender differences as confounding factors, we included only female donors. We included individuas with a BMI between 20 and 33. This population did not take any anti-inflammatory medication and their baseline C-reactive protein (CRP) and SAA levels were measured (Table 1). Blood was collected in citrated tubes by a qualified phlebotomist and left to stand for 30 minutes. Whole blood and platelet poor plasma were used in this study. Whole blood was kept at room temperature and analysed on the day of collection. Platelet poor plasma was derived by centrifuging whole blood for 15 minutes at 3000 *g* and stored at −80 °C. We also used pooled platelet poor plasma as one of our models. As positive control to show platelet activation and spreading, we include a figure from type 2 diabetes (T2DM); this is from raw data from a recently published paper^27^. This is how platelets look like in T2DM, which is a condition that is known to have platelets in a typical prothrombotic state - where both platelet hyperactivation and spreading occurs.

**Table 1:** Control donor data.

Demographic data (N = 21; all female)		
Attribute	Value	Reference Values (from Pathology Laboratory: Pathcare, Stellenbosch, South Africa)
Age (yrs)	48.05 ± 1.78	
Height (m)	1.69 [1.58–1.73]	
Weight (kg)	76.8 ± 4.78	
BMI (kg.m^−2^)	26.9 [20.55–33.65]	
GLU (mmol.L^−1^)	4.85 ± 0.13	3.5–5.5 mmol.L^−1^
TC (mmol.L^−1^)	5.48 ± 0.24	<5.0 mmol.L^−1^
HDL-C (mmol.L^−1^)	1.6 [1.35–1.75]	>1.0 mmol.L^−1^
LDL-C (mmol.L^−1^)	3.30 ± 0.19	<3.0 mmol.L^−1^
TG (mmol.L^−1^)	1.04 [0.85–1.74]	<1.70 mmol.L^−1^
Non-HDL (mmol.L^−1^)	3.92 ± 0.21	<3.8 mmol.L^−1^
TC:HDL	3.55 ± 0.14	<4.0
Ultra-sensitive CRP (mg.L^−1^)	2.5 ± 0.4	<1 mg. L^−1^ – low risk1–3 mg. L^−1^ – moderate risk >3 mg. L^−1^ – high risk >5.0 mg. L^−1^ – active infection/inflammation
SAA levels (µg.mL^−1^)	1.5 [0.7–2.2]	

Parametric measures are reported as mean ± SEM. Non-parametric data are reported as median [interquartile range]. Parameters assessed by the pathology laboratory also indicate the provided reference values. **BMI** = Body Mass Index; **GLU** = blood glucose; **TC** = total cholesterol; **HDL-C = **high-density lipoprotein cholesterol; **LDL-C = **low-density lipoprotein cholesterol; **TG** = triglycerides; **Non-HDL** = non-high density lipoprotein cholesterol; **TC:HDL** = total cholesterol to high density lipoprotein cholesterol ratio; **CRP** = C-reactive protein.

### Purification of human acute phase HDL (AP HDL), SAA and apoA-I

Blood for the purification of acute phase HDL was obtained at the University of Kentucky Medical Center, from patients post cardiac surgery, with informed consent (IRB 04-0218-P2J).

Human HDL containing SAA (known as acute phase HDL) was purified by sequential ultracentrifugation from plasma collected 24 hours after cardiac surgery as described^28^. SAA and apoA-I were purified from delipidated acute phase HDL by size-exclusion chromatography as described^29^.

### Product incubation with samples

The SAA levels of each of our healthy control donors were determined to confirm that they present in the normal range (Table 1). Purified SAA was added to donor platelet poor plasma (PPP) and whole blood (WB) *ex vivo* or to purified fibrinogen (Sigma, F3879) and FITC-labelled fluorescent fibrinogen (abcam, ab92811) *in vitro* at a final concentration of 30 or 100 μg.mL^−1^. This level is similar to that characterising a modest acute phase response^30^. A working solution of 0.166 and 2 mg.mL^−1^ purified fibrinogen was used and found to be a suitable concentration to form fibrin fibres in the presence of thrombin, similar to that of platelet-rich plasma fibres from healthy individuals^11^. ApoA-I (30 μg.mL^−1^) added to pooled PPP and purified fibrinogen was used as a negative control for the confocal experiment.

### Thromboelastography (TEG) with WB and PPP

A Thromboelastograph 5000 Hemostasis Analyzer System was used for viscometric clot property studies of whole blood (WB) and platelet poor plasma (PPP). WB and PPP were exposed for 10 minutes to SAA (30 μg.mL^−1^) before being analysed by TEG. 340 μL of exposed or naïve sample were added to 20 μL of 0.2 M CaCl_2_ in a disposable TEG cup to reverse the anticoagulant effect of the citrate. TEG assesses various kinetic clot parameters: i.e. R-time (clot initiation time, measured in minutes), Angle (the thrombin burst, measured as the slope between R and the 20 mm set point), MA (the maximum amplitude and the overall stability of the clot), MRTG (the maximum velocity of clot growth), TMRTG (the time of maximum rate of thrombin generation) and TTG (the total clot strength)^19,31–33^. This method is based on activation via the intrinsic clotting pathway upon re-calcification of a citrated anticoagulant sample, and contact activation between the manufacture-supplied cup and pin surface.

### Scanning Electron Microscopy (SEM) with WB and purified fibrinogen

SEM was used to study the ultrastructure of RBCs and platelets in whole blood, with a specific focus on their membranes. WB smears were prepared on the day of blood collection by pipetting 10 μL of the treatment groups (naïve or 30 μg.mL^−1^ SAA-exposed) onto a glass cover slip. After leaving to settle for 1 minute, preparation involved washing with PBS, 4% formaldehyde and 1% osmium tetroxide fixation steps, and dehydration in increasing grades of ethanol and 99.9% hexamethyldisilazane (HMDS) [for detailed methods see^19,34^]. Images were captured using a Zeiss Merlin (Gemini II) FE SEM. A purified fibrinogen sample without thrombin (i.e. no clot initiation) was also prepared for SEM analysis to determine if SAA causes the soluble fibrinogen proteins to form insoluble matted deposits.

### Confocal Microscopy with purified fibrinogen and FITC-fibrinogen

#### Purified fibrinogen

To study the protein structure and nature of purified fibrinogen upon exposure to SAA (30 μg.mL^−1^), we first imaged unstained samples, followed by the addition of amyloid-specific markers. The three fluorescent amyloid markers were prepared as follows: 5 µM thioflavin T (ThT), 0.1 µL (stock concentration as supplied) of Amytracker 480 and 0.1 µL (stock concentration as supplied) of Amytracker 680^9,10^ were added to the sample to incubate for 30 minutes. A working solution of Amytrackers was made in PBS at a 1:20 ratio. The exposure concentration of the working solution calculates back to 0.1 µL of stock solution as supplied.

Clots were made from the naïve or SAA-treated purified fibrin(ogen) by adding thrombin. Thrombin (South African National Blood Service) was solubilized in PBS containing 0.2% human serum albumin to obtain a concentration of 20 U.mL^−1^ and was used at a 1:2 ratio to create extensive fibrin networks. A coverslip was placed over the prepared clot, and samples were viewed using a Zeiss LSM 780 with ELYRA PS1 confocal microscope using a Plan-Apochromat 63x/1.4 Oil DIC objective. ThT was excited by the 488 nm laser^35^, with emission measured at 508 to 570 nm; Amytracker 480 was excited by the 405 nm laser, with emission measured at 478 to 539 nm; and Amytracker 680, was excited by the 561 nm laser, with emission measured at 597 to 695 nm. A selection of micrographs of the prepared clots was captured. Gain settings were kept the same for all data acquisition and used for statistical analyses; however, brightness and contrast were slightly adjusted for figure preparation.

#### FITC-fluorescent human fibrinogen

We added SAA (30 μg.mL^−1^) to FITC-fluorescent human fibrinogen, followed by thrombin, to create an extensive fibrin fibre network. FITC was excited at 488 nm, with emission measured at 508 to 570 nm. Amytracker 680 was added to FITC fibrinogen to visualise amyloid areas (emission and excitation as above).

### Confocal Microscopy with individual and pooled platelet poor plasma (PPP) clots

#### Individual donor PPP to detect amyloid

Individual platelet poor plasma (PPP) samples from healthy individuals were stored at −80 °C. On the day of analysis, the −80 °C stored PPPs were brought to room temperature. Naïve and SAA-exposed samples were prepared, with exposed samples incubated for 10 minutes with SAA. Here, all three fluorescent markers were added into the same sample. Thrombin was added (see above) to create extensive clots. The fluorescent signal of each of the three fluorescent markers was captured as a composite image file in the Zeiss ZEN software, whereupon ImageJ (FIJI) was used to split and analyse the RGB channels. The variance between (black) background and the presence of fluorescent pixels (binary comparison) were assessed for each of the three fluorescent markers in the clots. See^10,36^ for a detailed explanation of the methods. The histogram function in ImageJ (FIJI) was used to calculate the coefficient of variance (CV) (as SD/mean) of the histogram of different pixel intensities. This metric is used to quantify and discriminate between clots of naïve plasma and clots with added product.

#### Pooled PPP to detect amyloid and SAA (with SAA antibody)

SAA (30 μg.mL^−1^) was incubated with pooled healthy PPP for 30 minutes at room temperature. Amytracker 480 and 680 were added to the samples and incubated at room temperature for a further 30 minutes. Clot formation was initiated with thrombin and the clots ( ± SAA) were air-dried for 4 minutes. This was followed by fixation with 10% neutral buffered formalin (NBF). After phosphate-buffered saline (PBS) (pH = 7.4) washing steps, samples were blocked with 5% goat serum (in PBS), and incubated with anti-human SAA antibody (1:500 in goat serum) (Anti-Serum Amyloid A antibody [EP11592-92]; ab190802) for one hour. After further PBS washing steps, the sample was incubated with secondary antibody (1:500 in PBS) (Goat Anti-Mouse IgG H&L Alexa Fluor® 488; ab150113) at room temperature in the dark for a further hour. The samples were finally washed and a coverslip was mounted with a drop of Dako fluorescence mounting medium on a microscopy slide for confocal analysis. The prepared samples were viewed on a confocal microscope (details above). SAA-antibody was viewed at an excitation of 488 nm, with emission measured with a GaAsP detector at 493–630 nm. Amytracker signal was viewed as above.

### Fluorescent detection of amyloid signal in purified fibrinogen and pooled platelet poor plasma (PPP) after addition of apolipoprotein A-I (apoA-I)

Purified fibrinogen and pooled PPP were exposed to apoA-I (30 μg.mL^−1^) for 30 minutes followed by Amytracker 480 and 680 and ThT exposure for a further 30 minutes. Clot formation was initiated by thrombin, and the sample was visualised with a confocal microscope.

### Correlative light-electron microscopy (CLEM) to detect SAA (with SAA antibody detection) and amyloid (with Amytracker 680)

CLEM is a novel procedure where a confocal micrograph is correlated with a scanning electron microscope (SEM) micrograph^37–39^. For CLEM preparation, SAA (30 μg.mL^−1^) was added to purified fibrinogen, followed by Amytracker 680 exposure and clot formation by thrombin. SAA was identified with anti-SAA antibody staining as described above. The prepared sample was covered with PBS for super-resolution (confocal) analysis, and mounted in the Zeiss Shuttle-and-Find microscope sample holder. This sample holder has a marked coordinate system that can be calibrated with the sample and the instrument. Prior to imaging the sample, the microscope is calibrated to the sample holder. For improved resolution and subsequent correlation, the samples were imaged with the super-resolution structured illumination microscopy (SR-SIM) platform. For the SAA antibody SIM detection, the 488 nm 100 mW laser was used for excitation and emission was detected with a BP 495–550 filter and captured with an Andor EM-CCD camera iXon DU 885. For Amytracker 680, the 561 nm lasers were used for excitation, with the BP 570–620 emission filter. Z-stack micrographs were processed with the ZEN 2012 software, applying an optimised noise-filtering algorithm. Due to the multi-colour setup, a channel alignment correction was performed post-processing.

Following acquisition of the fluorescence micrographs, SEM sample preparation was immediately performed with the cover slip still mounted in the Shuttle and Find Holder. The sample was further fixed with 1% osmium tetroxide, then dehydrated using a standard series of ethanol dilutions: 30%, 50%, 70%, 90% and (3x) 100%. The sample was subsequently covered with 99.9% hexamethyldisilazane (HMDS) to complete sample dehydration and air dried in a fume hood overnight. The sample was coated with a thin (~5 nm) layer of carbon and imaged on a Zeiss MERLIN field emission scanning electron microscope at 1 kV using the Shuttle and Find modality to correlate areas of interest.

### Statistical analysis

TEG parameters were analysed by either the two-tailed paired t-test or the Wilcoxon matched-pairs test, after normality testing. This type of analysis allows us to compare each product exposure with the control (GraphPad Prism 7). Coefficient of variance (CV) from the fluorescent confocal data were analysed using Mann-Whitney test (GraphPad Prism 7).

### Data sharing

Raw data, including original images and micrographs without colour can be accessed at: https://1drv.ms/f/s!AgoCOmY3bkKHiOJ5yZeJLiYfzLq2yQ.

### Ethics approval and consent to participate

Ethical clearance was obtained from the Health Research Ethics Committee (HREC) of Stellenbosch University. A written form of informed consent was obtained from all donors. The Universities of Pretoria and Stellenbosch (clearance numbers UP355/2015, UP298/2016 and UP77/2017) approved this study. Written informed consent was obtained from all donors. Blood was collected and methods were carried out in accordance with the relevant guidelines of the ethics committees. We adhered strictly to the Declaration of Helsinki.

## Results

### SAA promotes atypical coagulation

The coagulation properties of platelet poor plasma (PPP) and whole blood (WB), with or without SAA treatment, are shown in Table 2. The thromboelastography (TEG) results for PPP reflect the effect of the added SAA on fibrin(ogen). The data indicate that SAA added at low concentrations (30 µg.ml^−1^) caused atypical coagulation: MA (increased clot density), MRTG (increased clot growth) and TTG (increased clot strength) were significantly changed. The TEG analysis of WB considers the participation of the formed elements in clot formation, namely platelets and RBCs, together with fibrin(ogen). TTG and MA were both significantly decreased, indicating a decreased clot strength with decreased platelet and/or fibrin(ogen) interaction, which results in a less dense clot that is also less rigid. The WB TEG results suggest a more flimsy clot, with few platelet interactions with (amyloidogenic) fibrin(ogen). Overall, the PPP and WB TEG results suggest a fibrin(ogen)-mediated enhancement of coagulation, but an inhibition of platelet/fibrin(ogen) interaction. These results point towards a dichotomous, complex interaction of SAA with fibrin(ogen) as well as the formed elements (platelets and RBCs) in blood.

**Table 2:** TEG analysis of platelet poor plasma (PPP) and whole blood (WB) before and after exposure to SAA.

TEG Parameters	Naïve PPP	+SAA	P Value: Naïve vs SAA
**PPP Analysis (n = 20)**			
R-Time	8.8 ± 1.6	8.3 ± 1.3	0.13
Angle	53.7 [51.3–56.0]	55.9 ± 6.9	0.09
MA	24.5 ± 6.2	26.0 ± 6.8	**0.03**
MRTG	3.5 [3.1–4.2]	4.2 [3.8–5.5]	**0.002**
TMRTG	10.1 ± 1.8	9.7 ± 1.6	0.17
TTG	168 ± 59	181 ± 65	**0.02**
**TEG Parameters**	**Naïve WB**	**+SAA**	**P Value: Naïve vs SAA**
**WB Analysis (n = 11)**			
R-Time	9.4 ± 2.6	8.9 ± 1.6	0.50
Angle	60.7 ± 4.8	60.6 ± 6.2	0.9
MA	61.6 ± 3.3	54.9 ± 7.0	**0.01**
MRTG	5.4 ± 1.0	5.2 ± 1.6	0.7
TMRTG	13.3 ± 3.5	12.7 ± 2.4	0.5
TTG	812 ± 110	628 ± 174	**0.009**

Parametric data are reported as mean ± SD, whereas non-parametric data are given as median and interquartile range (25% percentile – 75% percentile). Data pairs that are parametric were assessed using the Student paired t-test, whereas non-parametric data pairs where assessed using the Wilcoxon signed rank test. p < 0.05 is considered significant and are marked in bold^41^. **R-time**: reaction time (minutes); **Angle**: (degrees); **MA**: maximum amplitude (mm); **MRTG**: maximum rate of thrombus generation (Dyn.cm^−2^.s^−1^); **TMRTG**: time to maximum rate of thrombin generation (minutes); **TTG**: total clot strength (Dyn.cm^−2^).

### SAA promotes platelet activation but not extensive spreading

**Figure 1:**
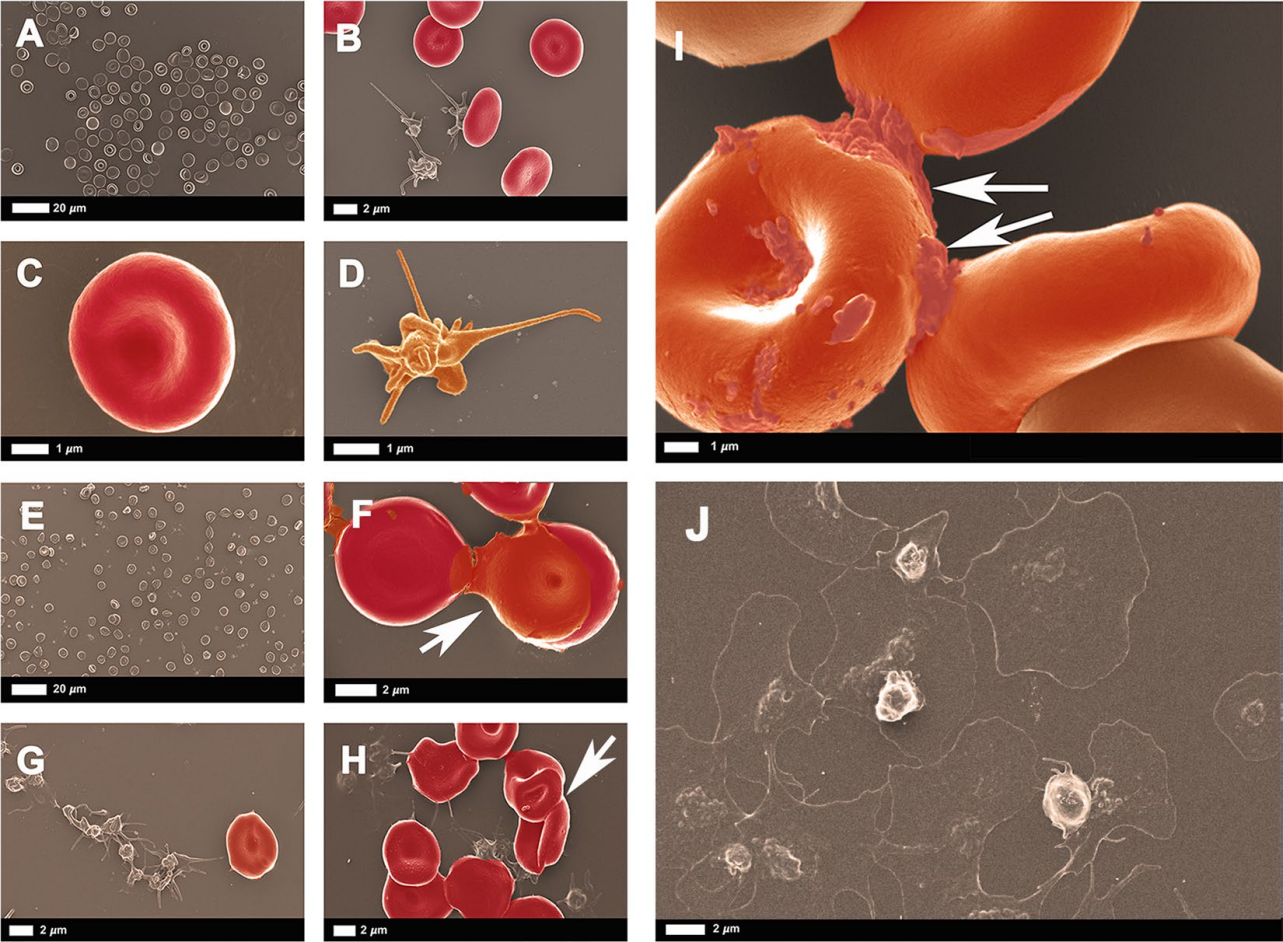
(**A**–**D**) WB smear prepared from healthy citrated blood. (**A**,**C**) Biconcave RBCs at different magnifications. (**B**) Platelets with slight pseudopodia formation. (**D)** Higher magnification of a representative healthy platelet with slight pseudopodia formation due to contact activation. (**E**–**I**) WB smear prepared from healthy citrated blood after exposure to SAA. WB incubated with 30 μg.mL^−1^ SAA show RBC agglutination **(E**,**F)** and activated and aggregated platelets (**G**,**H**). (**I**) WB incubated with 100 μg.mL^−1^ SAA reveals, at higher magnification, agglutinated RBCs with activated platelets; arrows indicate agglutination. (**J**) Example of platelet (hyperactivation) seen in type 2 diabetes (from raw data^27^). See scale bar below each micrograph.

The ultrastructure of RBC and platelets of healthy blood, before and after exposure to SAA is depicted in Fig. 1. Micrographs of naïve WB smears show the expected biconcave RBCs and slightly activated platelets with a few pseudopodia, due to contact activation (Fig. 1A–D). In Fig. 1E–I, micrographs of WB after exposure to 30 μg.mL^−1^ or 100 μg.mL^−1^ SAA, show RBC agglutination, platelet activation and increased platelet clumping but not platelet spreading. During a typical prothrombotic state, platelet activation and spreading result in an increased platelet/fibrinogen interaction. The platelets in this exposed WB sample show only enhanced pseudopodia formation in the presence of SAA, without spreading. We include a positive control (Fig. 1J) of platelets from type 2 diabetes, where platelets show both activation and spreading (raw data from a recently published paper^27^). This observation supports the TEG results, where a flimsy WB clot forms with compromised platelet/(amyloidogenic) fibrin(ogen) interactions. RBC agglutination will cause rheology impairment^40^, and provides evidence of both hypercoagulation and a pro-thrombotic state. Our raw dataset of micrographs can be accessed at the data sharing link given above.

### SAA promotes amyloid formation in purified and FITC-fibrin(ogen)

**Figure 2:**
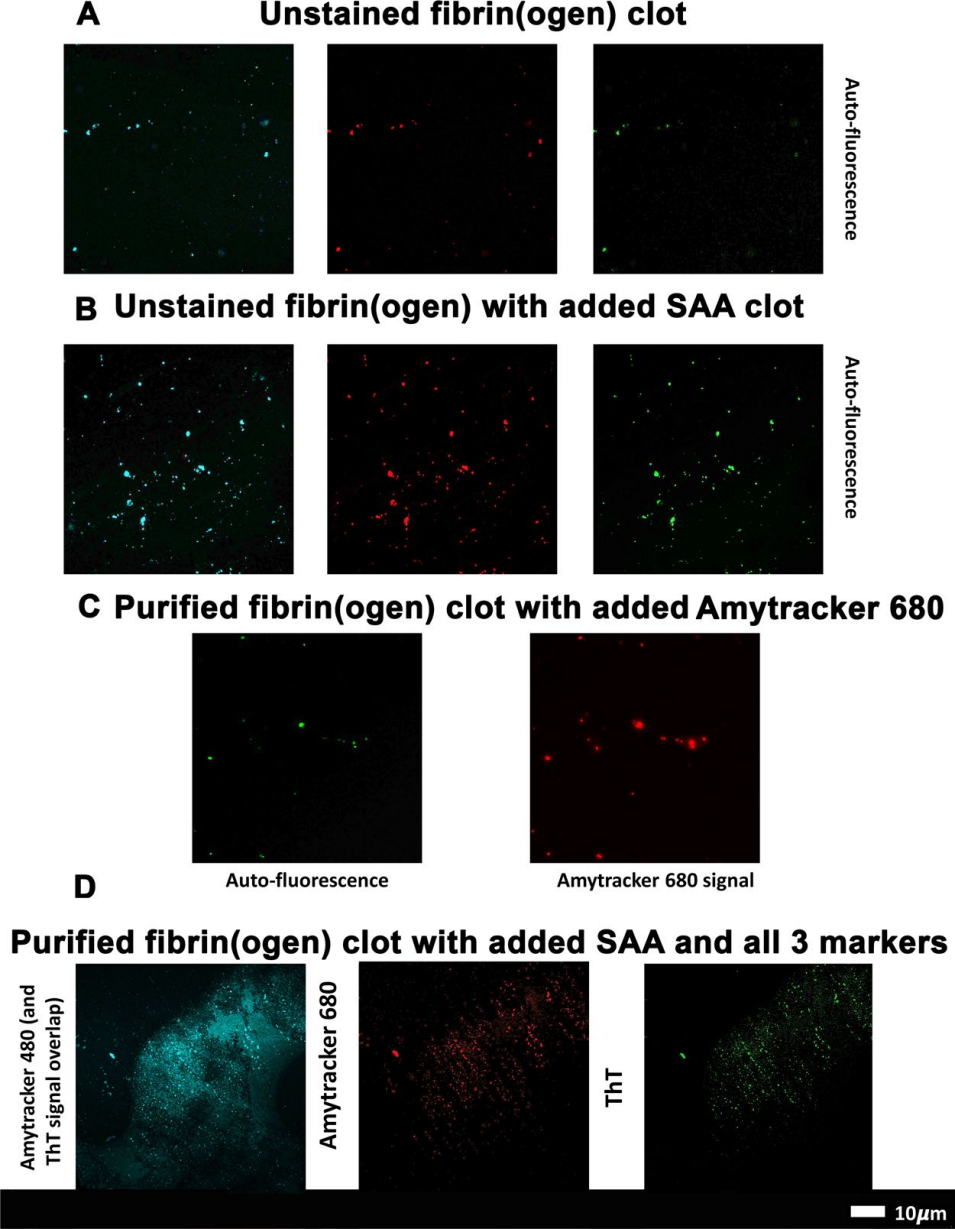
(**A**) Unstained purified fibrin(ogen) clots showing auto-fluorescent signal in all 3 marker channels. (**B**) Unstained purified fibrin(ogen) clots with added serum amyloid A, showing enhanced auto-fluorescent signal in all 3 marker channels. (**C**) Purified fibrin(ogen) clot with Amytracker 680. (**D**) Purified fibrin(ogen) clot with added SAA followed by Amytracker 480 (blue channel), 680 (red channel) and ThT (green channel).

Figure 2A,B depict confocal microscopy results of unstained purified fibrin(ogen) clots, with and without addition of 30 μg.mL^−1^ SAA. The unstained results suggest that there are areas of auto-fluorescence in purified fibrin(ogen) clots, and these areas can be picked up with the laser settings of the three amyloid markers. Figure 2C shows a purified fibrin(ogen) clot with added Amytracker 680 (single stain). Auto-fluorescence is detected in the green channel whereas amyloid areas are detected with Amytracker 680 in the red channel. The auto-fluorescent and amyloid signal share a similar amorphous morphology and appear to overlap in certain regions. Indeed, certain amyloid proteins have been shown to possess auto-fluorescent properties^42,43^, and investigating a potential correlation been the intrinsic optical properties of fibrinogen and (stained) amyloid signal presents a novel research question.

Figure 2D depicts confocal microscopy results, where purified fibrinogen was incubated with 30 μg.mL^−1^ SAA, together with all three fluorescent amyloid markers (Amytracker 480, 680 and ThT), and clotted with thrombin. Amytracker is newly developed specific amyloid stain with enhance optical properties, whereas ThT is a classical amyloid stain that binds to open hydrophobic areas that are considered equivalent to amyloid areas. ThT has a wide spectra where fluorescence can be detected^35^. Amytracker 480 and ThT therefore have similar excitation and emission spectra and we allowed these two stains, which both target amyloid structures, to overlap in the microscope setup to produce a combination (blue) channel of more pronounced amyloid signal, alongside the isolated signal from Amytracker 680’s red channel and the ThT’s isolated green channel.

**Figure 3:**
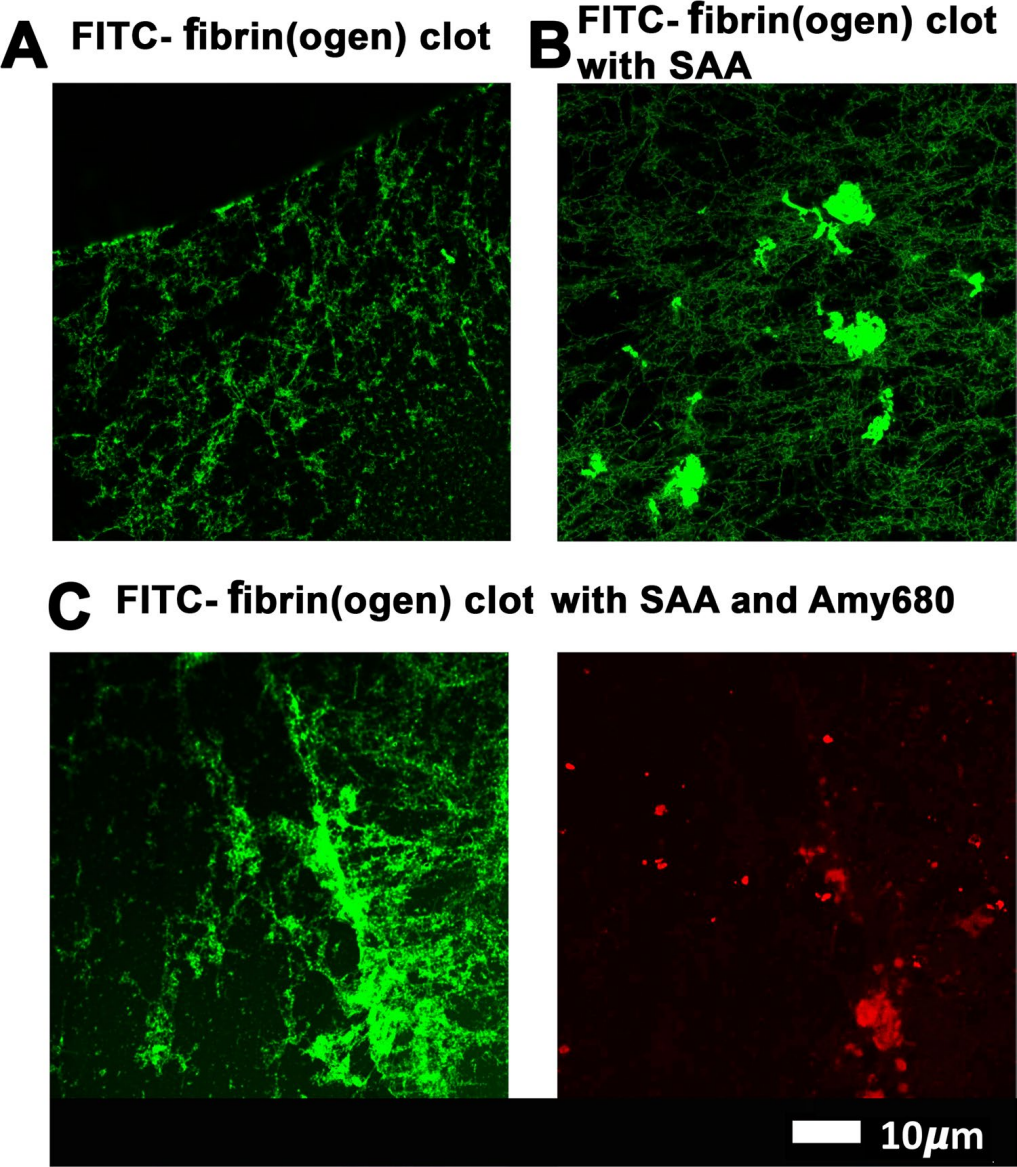
(**A**) FITC fibrinogen; (**B**) FITC fibrinogen with added SAA; (**C**) FITC fibrinogen with added SAA and Amytracker 680 (red).

Further to this, Fig. 3A,B show FITC-fibrin(ogen) clots, with and without SAA. Figure 3C shows a SAA-exposed FITC fibrinogen clot with added Amytracker 680. As was noted in purified fibrin(ogen) clots (Fig. 2C), naïve FITC-fibrin(ogen) clots also contain small areas of abnormal or misfolded protein. When SAA is added, larger areas of hypercoagulable fibrin(ogen) are noted in these clots. Amytracker 680 also binds in the same vicinity as the hypercoagulable areas. These results point to the fact that anomalous hypercoagulated areas, that form in the presence of SAA, have an amyloid nature. These results are in line with previous findings, where we showed that increased amyloid is present in fibrin(ogen) in conditions such as Type 2 Diabetes, Parkinson’s Disease and Alzheimer Disease, where hypercoagulation is well-known^44–47^.

Finally, even though SAA is an amyloid protein itself, it does not show fluorescence when stained alone with amyloid markers (data not shown). We therefore suggest that the amyloid signal comes from the fibrinogen, the major protein in PPP, that changes in the presence of SAA to become hypercoagulated and that these hypercoagulated areas are indeed amyloid in nature. These results suggest that SAA, when added in a purified form, can induce amyloidogenesis of both purified and FITC-fibrin(ogen). We next quantitatively investigate the influence of SAA on healthy plasma, as well as confirm SAA interaction with fibrinogen by antibody binding and correlative microscopy.

### SAA induces amyloid fibrils in individual healthy plasma clots

**Figure 4:**
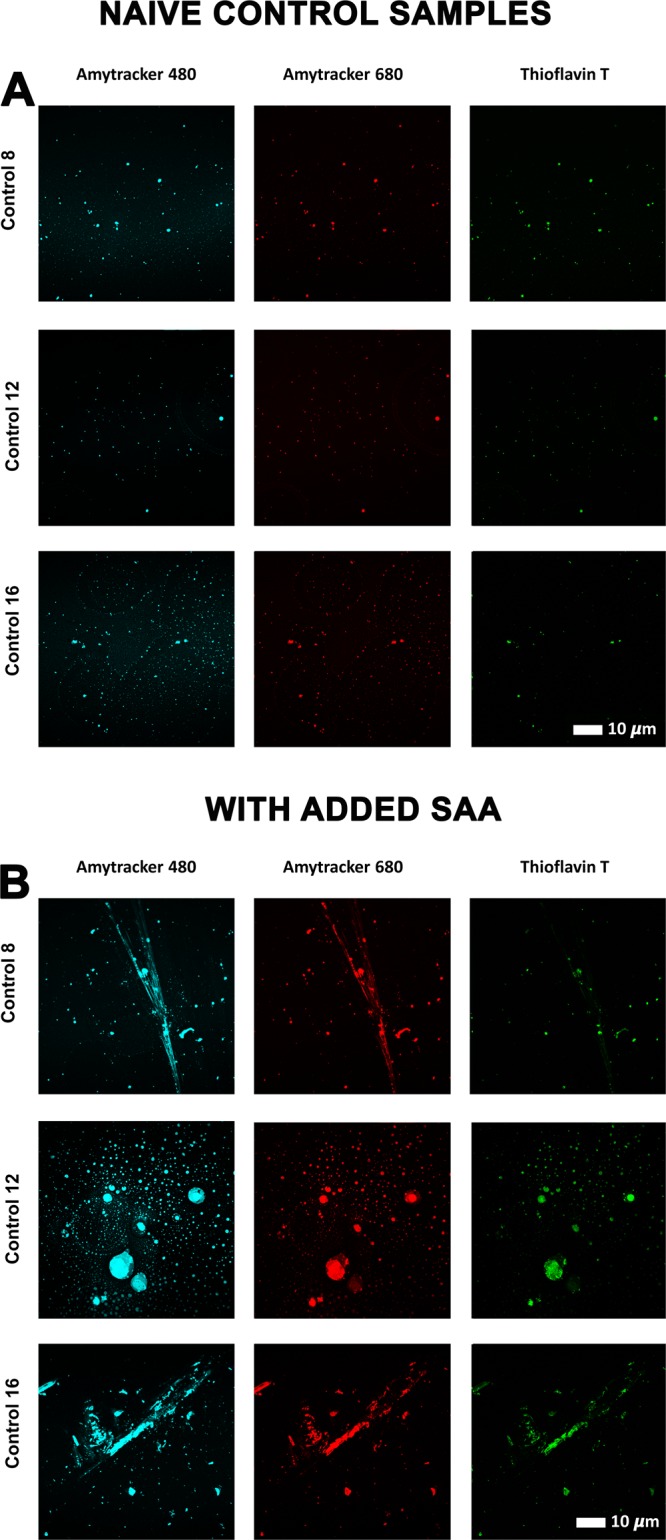
(**A**) Fluorescent signals from amyloid-selective markers of thrombin-induced PPP clots from three representative naïve control donors. (**B**) Fluorescent signals from amyloid-selective markers of thrombin-induced PPP clots from three control donors exposed to (30 μg.mL^−1^) SAA. (The blue channel represents signal from both Amytracker 480 and ThT).

**Figure 5:**
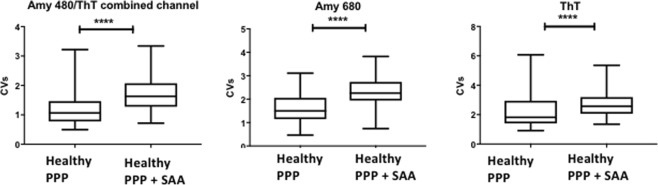
Coefficient of variation (CV) analysis of confocal microscopy micrographs. Fluorescent results from samples with the three fluorescent amyloid markers: Amytracker 480 ThT combined channel, Amytracker 680 and Thioflavin-T are presented as boxplots. Samples are naïve PPP or PPP incubated with 30 μg.mL^−1^ SAA. P-values for all three markers were < 0.0001 (****) (Mann-Whitney analysis); Coefficient of Variation (CV).

Individual healthy PPP (n = 19) was incubated without or with 30 μg.mL^−1^ SAA, followed by exposure to the 3 amyloid fluorescent markers and clot preparation. Figure 4A,B represent fluorescent signals of the amyloid markers under the different exposure conditions. Very little amyloid signal is seen in healthy naïve clots (Fig. 4A), while added SAA caused a marked increase in all 3 fluorescent marker signals (p < 0.0001), with large patches of visible amyloid (Fig. 4B). This observation provides evidence that the presence of free SAA circulating at the level present in a modest acute phase response can induce atypical clotting. Figure 5 represents the coefficient of variance (CV) data for the pixel intensities of the confocal microscopy data. Multiple micrographs were captured for each sample. In total, 94 control and 96 SAA micrographs were analysed.

### SAA is detected with antibody binding in the same areas as amyloid signal, in both pooled plasma and purified fibrinogen

**Figure 6:**
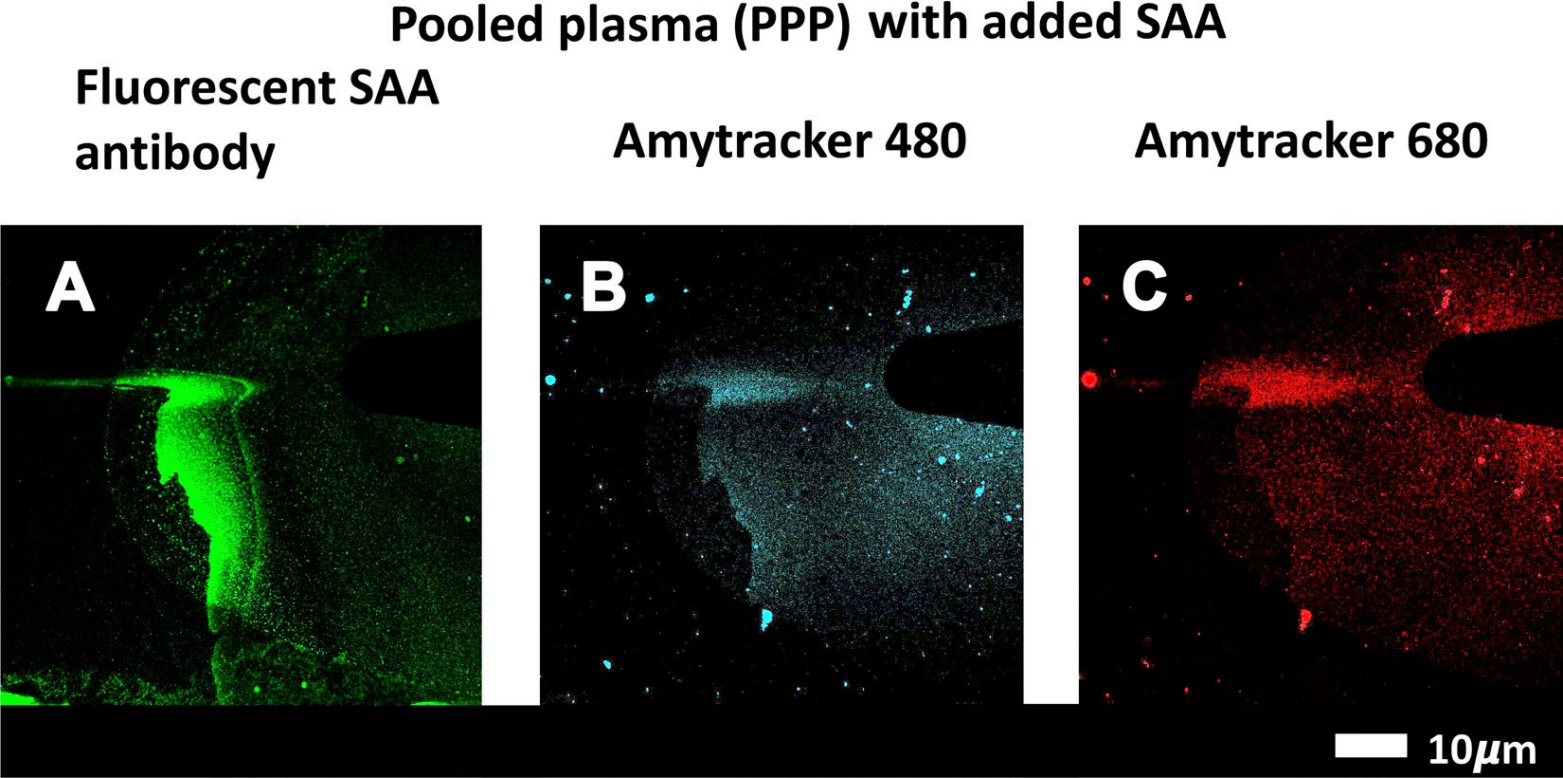
Pooled PPP incubated with 30 μg.mL^−1^ SAA followed by exposure to (**A**) fluorescent SAA antibody, (**B**) Amytracker 480 and (**C**) Amytracker 680, and clotted with thrombin.

To confirm that an interaction between SAA and the fibrin(ogen) protein is present, a pooled PPP smear was stained with anti-SAA fluorescent antibody, followed by addition of Amytracker 480 and 680 (Fig. 6). SAA could be detected with its antibody and amyloid signal was detected in overlapping areas to the SAA antibody.

**Figure 7:**
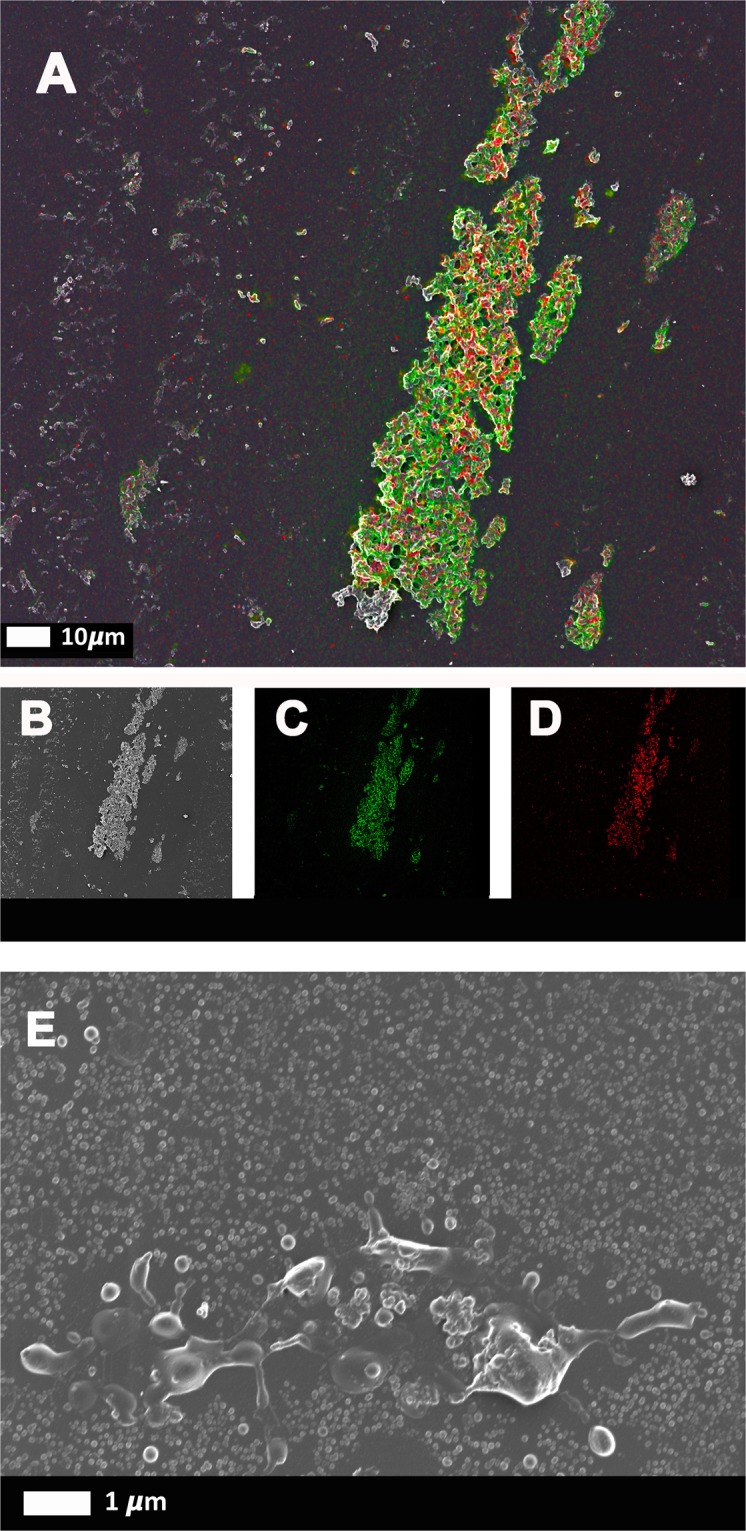
Correlative Light Electron Microscopy (CLEM) confirms the presence of SAA in amyloidgenic fibrinogen clots. Purified fibrinogen was incubated with 30 μg.mL^−1^ SAA, and marked with Amytracker 680 and fluorescent SAA antibody. (**A**–**D**) Fibrinogen clotted with thrombin. (**A**) Super-resolution micrograph combined with SEM micrograph using CLEM technology. (**B**) SEM micrograph. **(C)** Fluorescent SAA antibody micrograph. **(D)** Fluorescent Amytracker 680 micrograph. **(E)** SEM image of purified fibrinogen with SAA, but without thrombin (not clotted).

Furthermore, correlative microscopy of a purified fibrinogen clot stained for SAA with antibody and for amyloid with Amytracker 680 confirms that SAA binds to purified fibrinogen, where it can provide a stimulus for the protein structure to become amyloidogenic. In Fig. 7A, the green antibody fluorescence is detected in close proximity to the red (Amytracker 680) amyloid fluorescent signal. Figure 7B represents the SEM analysis, Fig. 7C the SAA antibody fluorescence and Fig. 7D the Amytracker 680 fluorescence. Figure 7E shows a SEM micrograph of purified fibrinogen with added SAA, but without thrombin. These results also confirm that SAA causes soluble fibrinogen to polymerise into insoluble matted deposits. Incubation of purified fibrinogen with human apoA-I, at 30 μg.mL^−1^ for 30 minutes, followed by incubation with Amytracker and thrombin did not yield a fluorescent amyloidogenic signal (data not shown).

## Discussion

Intimate associations exist between inflammation and thrombosis, with the inflammatory state promoting coagulation^7^. These processes share key molecular mechanisms and can be viewed as intrinsically linked processes^48,49^. HDL serves as a modulator of platelet and coagulation responses^50^. However, inflammatory HDL is remarkably remodelled by acute phase SAA associating with it, to the extent that SAA can even become the major apolipoprotein^28^. This SAA, that can increase up to a thousand-fold in plasma, has been shown to impact platelet adhesion and activation^25^. However, it should be kept in mind that this SAA is not free, but mostly bound to HDL.

Here we established that free SAA, added at low physiological levels (30 µg.mL^−1^), representing a modest acute phase response, caused red blood cell agglutination, platelet activation and aggregation, but without platelet spreading. Platelet spreading indicates increased activation where the platelet loses its rigid structure, a process whereby adherent platelets will flatten at sites of vascular injury, with increased deformation of its membrane. It is plausible that the highly lipophilic SAA interacts within the platelet cell surface phospholipids (analogous to HDL) to produce these effects.

In previous work, we demonstrated that exceptionally low (and highly substoichiometric) concentrations of LPS, as well as LTAs, could induce the formation of an amyloid form of fibrin^10^. This novel finding supported previous research by Strickland and colleagues that showed that fibrin(ogen) can interact with known amyloid-forming peptides and proteins^22,24,51–54^. We established here that SAA (30 µg.mL^−1^) can similarly induce amyloid fibrin fibrils that could affect clot formation, likely in an interactive manner. We added SAA (30 µg.mL^−1^) to purified fibrinogen, FITC-fibrinogen and to individual and pooled donor PPP, both with and without thrombin. We confirmed SAA binding to purified fibrinogen and to pooled PPP with the use of fluorescent SAA antibody binding (Figs 6 and 7). We also showed that amyloid can be induced in purified fibrinogen, after exposure to SAA, and that SAA binding occurs in the same areas as the amyloid areas on fibrin(ogen), using CLEM technology. SAA added to purified fibrinogen also formed spontaneous dense and matted fibrin(ogen) deposits, without addition of thrombin (Fig. 7E). These results therefore suggest that circulating SAA might bind to soluble and circulating plasma molecules, such as fibrinogen, and cause structural (amyloidogenic) changes. These findings support our WB analysis, where we showed, that after addition of SAA, spontaneous plasma deposits were noted that caused RBCs to become agglutinated to each other (see Fig. 1; arrows). Incubation of purified fibrinogen with the apolipoprotein apoA-I (30 µg.mL^−1^) did not yield a fluorescent amyloid signal with Amytrackers.

A further interesting finding was that hypercoagulated areas (or anomalous clotted fibrin deposits) show auto-fluorescence in purified and FITC fibrin (ogen), as well as pooled plasma clots. Autofluorescence was previously reported in fibrin microbeads, prepared from native and heat denatured fibrinogen, activated by thrombin and crosslinked by endogenous factor XIII^55^. Furthermore, these areas bear similarities to the areas where our amyloid markers also bind. This is in line with previous findings that suggest that an intrinsic fluorescence, in the visible range, develops during the aggregation of a range of polypeptides, including the disease-related human peptides amyloid-β^42,43^.

A notable aspect of our results is that all the plasma tested contained normal levels of HDL. SAA readily associates with HDL, but our findings indicate that sufficient “free” SAA is available to affect platelet activation and clot formation. It is this “free” (non HDL-associated) SAA that has been shown to be biologically active^56^. HDL apolipoproteins (apoA-I, SAA) exist in a dynamic equilibrium between the HDL-bound and free form^57^. During the acute phase response, HDL is drastically remodeled by the combined action of cholesterol ester transfer protein (CETP) and the acute phase induced group II secretory phospholipase A2 (sPLA2)^28^. The action of CETP on the core of the particle and sPLA2 hydrolysis of the HDL surface synergises the liberation of lipid-poor apoA-I and SAA. It is this free SAA that can deposit as reactive systemic amyloid fibrils during chronic inflammatory conditions. The lipid-poor or free SAA fraction during inflammation is thus a function of these remodeling activities. As an exchangeable apolipoprotein, a non-HDL SAA concentration of 30–50 ug∙mL^−1^ is readily achievable, even during modest inflammation^58^.

In addition to the huge induction of SAA during inflammation that alters HDL apolipoprotein composition, acute phase phospholipases are also induced^59,60^. They hydrolyse HDL surface phospholipids, accelerating HDL metabolism and impacting the ratio of HDL-associated and “free” SAA towards the latter, potentially increasing the pro-inflammatory and pro-coagulant form of SAA^28,56^. The SAA-enriched acute-phase HDL would also interact with the classical HDL receptor scavenger receptor class B type I (SR-BI) resulting in selective lipid uptake from the particle^61^. SR-BI remodeling of acute-phase HDL could additionally propel SAA to a “free” form^28^. In addition, SR-BI is present on platelets where it can modulate platelet reactivity and thrombosis in dyslipidemia^62^.

In the present paper, we focused only on the effects of free SAA added *ex vivo*. However, we do appreciate that there are many physiological factors including the simultaneous presence of e.g. lipases, and factors such as blood rheology, that play important roles. Here, we confirmed that SAA added at low physiological concentrations (30 μg.mL^−1^) caused an increase in fluorescent staining in PPP, as seen by confocal microscopy, suggesting the formation of pathological amyloid fibrin.

The TEG, SEM and confocal amyloid marker results from both WB and PPP analyses point collectively to the development of a pro-coagulant state when free SAA is present. The formed clot is more fragile with possibly compromised platelet interactions. Overall our results suggest a fibrin(ogen)/SAA-mediated enhancement of coagulation, but an inhibition of platelet/fibrin(ogen) interactions; with this interaction potentially including amyloid fibrin(ogen). This is the result of a dichotomous complex interaction of SAA with fibrin(ogen) as well as the formed elements (viz. platelets and RBC) in blood. In this study, we have not investigated the mechanism by which SAA might exert its effects. However, mechanistically, SAA could inhibit platelet activities such as degranulation, expression of GPIIb/IIIa, or contraction via microtubules. This would prevent the usual platelet-fibrin polymer interaction. Additionally, changes in the fibrin structure to an anomalous amyloid form may also affect this interaction.

A clearer and more mechanistic definition of SAA impacting coagulation during inflammation is essential to understand better its value in diagnosis and even therapy. This would be complex *in vitro* given the large number of enzymes and receptors that affect HDL and SAA during this process. To explore these questions *in vivo*, animal models would be required where SAA genes are deleted or SAA is over-expressed with viral vectors^5,63^. Overall, the results presented in this paper demonstrate that SAA can directly bind to fibrin(ogen), and that SAA can affect coagulation by promoting amyloid formation in fibrin(ogen), as well as influencing platelets to be more prothrombotic.
